# Supplementary material for: A general mechanism of KCNE1 modulation of KCNQ1 channels involving non-canonical VSD-PD coupling
Source: Commun Biol. 2021 Jul 20;4:887. doi: 10.1038/s42003-021-02418-1 (PMC8292421; doi:10.1038/s42003-021-02418-1)
Supplement: Supplementary file 1 — Supplementary information. [file 42003_2021_2418_MOESM1_ESM.pdf]

A general mechanism of KCNE1 modulation of KCNQ1 channels involving non-canonical VSD-PD coupling

Xiaoan Wu[1], Marta E. Perez[1], Sergei Yu Noskov[2], H. Peter Larsson[1]*

[1] Department of Physiology and Biophysics, Miller School of Medicine, University of Miami, Miami, FL 33136, USA.

[2] Centre for Molecular Simulation, Department of Biological Sciences, University of Calgary, Calgary, AB, Canada, T2N 1N4

* H. Peter Larsson

Email: plarsson@med.miami.edu

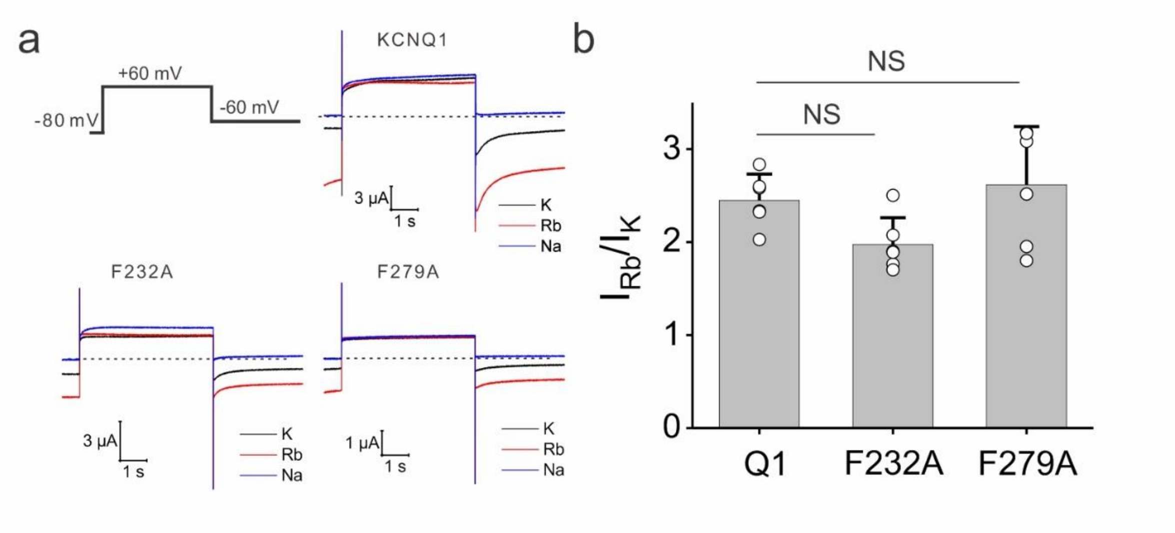

Supplementary figure 1. F232A and F279A do not change the Rb^+/K^+ permeability ratio of KCNQ1 channels in the absence of KCNE1. **(a)** Current traces from oocytes expressing KCNQ1, KCNQ1-F232A and KCNQ1-F279A channels under high external K^+ (black), Rb^+ (red) and Na^+ (blue) concentration. Dashed lines indicate zero currents. Cells are held at -80 mv and stepped to +60 mV followed by a step to -60 mV. Note that KCNQ1 channels do not close completely at negative voltages. **(b)** Comparison of measured tail Rb^+/K^+ ratio from the KCNQ1 (n=6), KCNQ1-F232A (n=6) and KCNQ1-F279A (n=6) channels. Data are shown as mean ± SEM, P=4.09349E-10 between KCNQ1 and KCNQ1/KCNE1 channels. P= 0.0002 between KCNQ1/KCNE1 and KCNQ1-F279A/KCNE1 channels, NS indicates no significant difference.

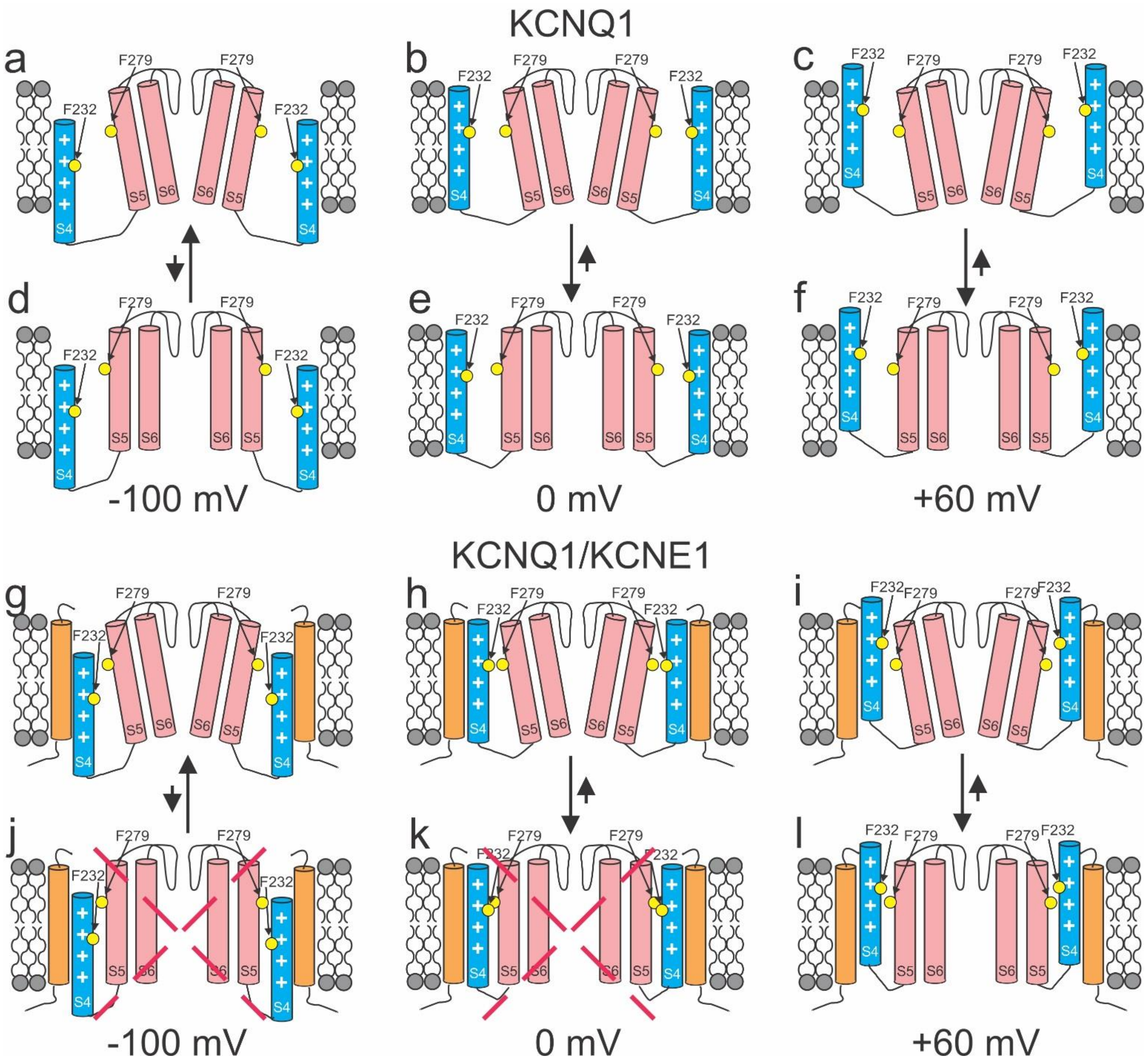

Supplementary figure 2. KCNE1-induced rotation of the VSD changes the VSD-PD coupling of KCNQ1. Schematic side view of KCNQ1 channels at resting-closed **(a)**, intermediate-closed **(b)**, activated-closed **(c)**, resting-open **(d)**, intermediate-open **(e)**, and activated-open **(f)** states. Schematic side view of KCNQ1 channels in the presence of KCNE1 at resting-closed **(g)**, intermediate-closed **(h)**, activated-closed **(i)**, resting-open **(j)**, intermediate-open **(k)**, and activated-open **(l)** states. Resting-open and intermediate-open state are suppressed in KCNQ1/KCNE1 channels. The clash between F232 and F279 inhibits the opening of KCNQ1 channels in the presence of KCNE1 when S4 is in the intermediate state. Residues responsible for KCNE1 suppressing the RO state of KCNQ1 channels are still unclear. Only S4 (blue), S5 and S6 (pink) are shown for clarity.

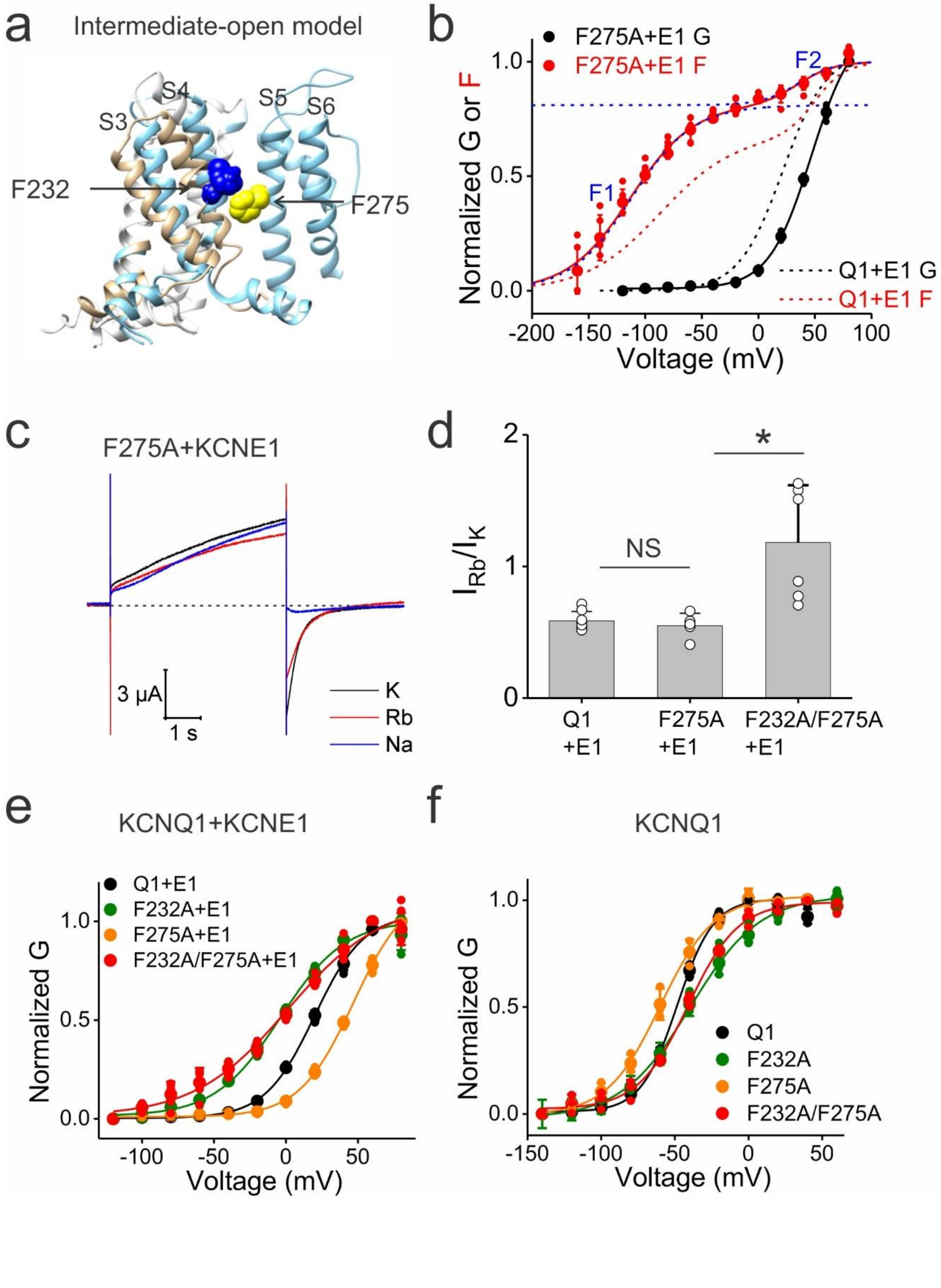
a
Intermediate-open model
S3
S4
S5
S6
F232
F275
b
F275A+E1 G
F275A+E1 F
F1
F2
Q1+E1 G
Q1+E1 F
Normalized G or F
Voltage (mV)
c
F275A+KCNE1
3 μA
1 s
K
Rb
Na
d
NS
*
I_Rb/I_K
Q1 +E1
F275A +E1
F232A/F275A +E1
e
KCNQ1+KCNE1
Q1+E1
F232A+E1
F275A+E1
F232A/F275A+E1
Normalized G
Voltage (mV)
f
KCNQ1
Q1
F232A
F275A
F232A/F275A
Normalized G
Voltage (mV)

Supplementary figure 3. F275A does not allow KCNQ1/KCNE1 to open in the intermediate S4 state. (a) F232 (blue) in S4 is close to F275 (yellow) in the intermediate-open model. **(b)** Voltage dependence of currents (black) and fluorescence (red) from KCNQ1/KCNE1 (dashed lines for comparison) and KCNQ1-F275A/KCNE1 (circles) channels. F1 and F2 indicate the first and second fluorescence components from KCNQ1-F275A/KCNE1 channels (blue dashed lines), respectively. **(c)** Current traces from oocytes expressing KCNQ1-F275A/KCNE1 channels under high external K^+ (black), Rb^+ (red) and Na^+ (blue) concentration in response to voltage protocols indicated in Figure 3. The dashed line indicates zero currents. **(d)** Comparison of measured tail Rb^+/K^+ ratio from the KCNQ1/KCNE1 (n=8), KCNQ1-F275A/KCNE1 (n=5) and KCNQ1-F232A-F275A/KCNE1 (n=6) channels. **(e)** Voltage dependence of current activation from wt KCNQ1+KCNE1 (black, n=4), KCNQ1-F232A/KCNE1 (green, n=4), KCNQ1-F275A/KCNE1 (orange, n=4) and KCNQ1-F232A-F275A/KCNE1 (red, n=6) channels. **(f)** Voltage dependence of current activation from wt KCNQ1 (black, n=4), KCNQ1-F232A (green, n=3), KCNQ1-F275A (orange, n=3) and KCNQ1-F232A-F275A (red, n=3) channels without KCNE1 association. Data are shown as mean ± SEM. * $P<0.05$, NS indicates no significant difference. P=0.4552 between KCNQ1/KCNE1 and KCNQ1-F275A/KCNE1 channels. P=0.0119 between KCNQ1-F275A/KCNE1 and KCNQ1-F232A-F275A/KCNE1.

	$G_{1/2}$ (mV)	n	$F1_{1/2}$ (mV)	$F2_{1/2}$ (mV)	n
Wt KCNQ1	-49.30 ± 1.39	4	-47.88 ± 0.68	58.30 ± 15.57	4
F232A	-39.40 ± 1.61	3	-40.07 ± 1.68	48.95 ± 3.24	3
F279A	-57.13 ± 2.96	4	-56.51 ± 2.23	36.37 ± 5.78	3
Wt KCNQ1/KCNE1	21.58 ± 0.56	4	-86.51 ± 4.49	50.14 ± 3.52	5
KCNQ1-F232A/KCNE1	-2.57 ± 1.61	4	-97.40 ± 1.65	49.94 ± 4.82	3
KCNQ1-F279A/KCNE1	-18.56 ± 1.65	3	-61.20 ± 10.59	39.27 ± 3.88	3
KCNQ1-F275A/KCNE1	46.77 ± 0.92	4	-112.99 ± 1.65	35.34 ± 10.17	4

Supplementary table 1. Summary of parameters for KCNQ1 and KCNQ1/KCNE1 mutant channels. $G_{1/2}$ is the voltage where 50% of the maximal conductance level is reached. $G_{1/2}$ was obtained by fitting the GV relation with a single Boltzmann equation. $F1_{1/2}$ is the voltage where 50% of the first component of fluorescence is reached and $F2_{1/2}$ is the voltage where 50% of the second component of fluorescence is reached. $F1_{1/2}$ and $F2_{1/2}$ were obtained by fitting the FV relation with a double Boltzmann equation. Data are shown as mean ± SEM, n indicates the number of replicates of all experiments.

Supplementary Data 1. Coordinates of the KCNQ1/KCNEB1 IO model in PDB format.
