# Supplementary material for: A general mechanism of KCNE1 modulation of KCNQ1 channels involving non-canonical VSD-PD coupling
Source: Commun Biol. 2021 Jul 20;4:887. doi: 10.1038/s42003-021-02418-1 (PMC8292421; doi:10.1038/s42003-021-02418-1)
Supplement: Supplementary file 5 — Description of Supplementary Files. [file 42003_2021_2418_MOESM5_ESM.pdf]

Description of Additional Supplementary Files

File name: Supplementary Data 1
Description: Coordinates of the KCNQ1/KCNEB1 IO model in PDB format.

File name: Supplementary Data 2
Description:

Fig. 2b
Effects of F232A on the GV and FV relations of KCNQ1/KCNE1 channels
Fig. 3b
Comparison of measured tail Rb+/K+ ratio from the KCNQ1, KCNQ1/KCNE1 and KCNQ1-F232A/KCNE1 channels
Fig. 4b
Effects of F279A on the GV and FV relations of KCNQ1/KCNE1 channels
Fig. 4d
Comparison of measured tail Rb+/K+ ratio from KCNQ1, KCNQ1/KCNE1 and KCNQ1-F279A/KCNE1 channels
Fig. 5b
Effects of F232A on the GV and FV relations of KCNQ1 channels
Fig. 5c
Effects of F279A on the GV and FV relations of KCNQ1 channels
Fig. 6c
GV relations from KCNQ1/KCNE1, KCNQ1-F232A/KCNE1, KCNQ1-F279A/KCNE1 and KCNQ1-F232A-F279A/KCNE1 channels
Fig. 6d
GV relations from KCNQ1, KCNQ1-F232A, KCNQ1-F279A and KCNQ1-F232A-F279A channels
Fig. 8b
GV relations from KCNQ1/KCNE1, KCNQ1-F232A/KCNE1, KCNQ1-L271A/KCNE1 and KCNQ1-F232A-L271A/KCNE1 channels
Fig. 8c
GV relations from KCNQ1, KCNQ1-F232A, KCNQ1-L271A and KCNQ1-F232A-L271A channels
Fig. S1
Comparison of measured tail Rb+/K+ ratio from the KCNQ1, F232A and F279A channels
Fig. S3b
Effects of F275A on the GV and FV relations of KCNQ1/KCNE1 channels
Fig. S3d
Comparison of measured tail Rb+/K+ ratio from the KCNQ1/KCNE1, KCNQ1-F275A/KCNE1 and KCNQ1-F232A-F275A/KCNE1 channels
Fig. S3e
GV relations from KCNQ1/KCNE1, KCNQ1-F232A/KCNE1, KCNQ1-F275A/KCNE1 and KCNQ1-F232A-F275A/KCNE1 channels
Fig. S3f
GV relations from KCNQ1, KCNQ1-F232A, KCNQ1-F275A and KCNQ1-F232A-F275A channels
